# Effects of duo-strain probiotics on growth, digestion, and gut health in broiler chickens

**DOI:** 10.1016/j.vas.2024.100343

**Published:** 2024-03-02

**Authors:** Seyed Mehrdad Mirsalami, Mahsa Mirsalami

**Affiliations:** a Department of Chemical Engineering, Faculty of Engineering, Islamic Azad University Central Tehran Branch, Tehran, Iran; b Faculty of Engineering and Technical Sciences, Qazvin Islamic Azad University, Qazvin, Iran

**Keywords:** Duo-strain probiotics, Broiler, Gut Microbiome, Antioxidant levels, Growth rate, Enzymatic digestion

## Abstract

The goal of this inquiry was to analyze the impact of incorporating *Enterococcus faecium* and *Streptococcus thermophilus* using a novel premix-spray method on the following aspects: growth rate, digestive enzyme activity, antioxidant levels, gut microbiome composition, and the morphological characteristics of the duodenum, jejunum, and ileum in broiler chickens. Furthermore, this study explored the potential benefits of duo strains of probiotics (DSP) in reducing flatulence, regulating stool microbial population, and improving diarrhea symptoms. A total of 360 one-day-old mixed-sex Plymouth Rock chicks (IW: 51 ± 33 g) were randomly divided into two treatment groups. Each treatment group was further divided into 9 replicated cages, with 20 chicks housed in each cage. The control group (CG) received a basal diet composed of a soy-corn mixture, whereas the experimental group was provided with DSP (CON + 0.5 % probiotic). The results showed that the increase in the body weight of broilers at the end of the fourth week in the control group and the treatment group was 1.576 versus 1.847 kg, respectively. Throughout the 30-day trial period, the DSP diet significantly improved the specific growth rate (SGR), survival rate (SR), and body weight gain (BWG) while decreasing the feed conversion ratio (FCR) (*P* < 0.05). The DSP diet also enhanced the Enzymatic digestion (protease, amylase, lipase, and trypsin) and antioxidant potential (SOD, MDA, and catalase) of the broilers compared to those in the CG. The results revealed significant enhancements in the tissue morphology of the duodenum and jejunum following the combined treatment for a duration of 4 weeks. The DSP treatments significantly increased microvillus height in the duodenum and jejunum but had no notable effects in the ileum. Incorporating 0.5 % DSP in poultry feed improved the relative abundance of *Ruminococcaceae* and *Faecalibacteriumin*, leading to better management of diarrhea and reduced presence of *E. coli* compared to the control diet. Additionally, including probiotics in the basal diet reduced H_2_S, CO_2_, NH_3_, and CH_4_ levels. Overall, the study suggests that the new spray-drying approach with these strains has potential for supplementing probiotics in poultry feed processing, and including DSP in broiler chicken diets has beneficial effects.

## Highlights

• A duo-strain probiotic (DSP; *E. faecium* and *S. thermophilus*) could improve growth, health, and beneficial native microbiota abundances of broilers.
• Spray-drying technology could maintain the high alive cells of probiotics.
• DSP strains are resistant to high temperatures.
• DSP strain is an effective approach in inhibiting pathogenic bacteria intestinal tract.
• Addition of probiotics to animal feed led to the treatment of diarrhea and weight gain.

## Introduction

The Plymouth Rock breed is one of the best breeds of broiler chickens for the production of chicken meat all over the world and its production shows a continuous upward trend, comprising over a quarter of the overall global aviculture yield (Guo et al., 2019). However, as aviculture progresses, a range of concerns pertaining to poultry production have emerged. For example, the prolonged misuse of antibiotic medications has resulted in a growing resistance among poultry pathogens, such as the infectious Bronchitis virus, inflicting serious harm to the security of domestic poultry products and the quality of chicken meat (Archacka et al., 2020; da Costa et al., 2018); when used commonly with animal feed results in a reduction in the resilience to diseases and increases the antimicrobial resistance of broiler (Sugiharto et al., 2022); disregard for proper air ventilation, resulting in the degradation of air quality and a rise in diseases outbreaks of Avian Influenza and Infectious Bronchitis virus (Mirsalami & Mirsalami, 2024b). The expansion, resistance, and fatality of broilers are interconnected issues that have significant impacts on the safety of poultry feed, as well as the productivity and profitability of aviculture.

According to the statistics of WHO and FAO, probiotics are regarded as a promising, secure, and environmentally friendly substitute for antibiotics, which are extensively utilized as feed supplements in aviculture, improving growth, facilitating nutrient absorption, bolstering host immunity, balanced gut microbiota, and improving poultry rearing environment (de Marins et al., 2022). *Lactobacillus acidophilus, Bifidobacterium spp, Propionibacterium, Aspergillus,* and *Bacillus spp* are among the most frequently used probiotics in poultry farming and are commonly included in poultry feed supplements (Woyengo et al., 2023). However, the production and preparation of these probiotics have become increasingly challenging due to rising demand, higher costs, and limited resources. As a result, there is a growing need to develop innovative and cost-effective approaches to produce probiotics with new strains to ensure their availability for use in poultry farming. Two lesser-known probiotic strains, *Streptococcus thermophilus,* and *Enterococcus faecium*, have recently been studied for their potential benefits in poultry production. These specific probiotic strains have been demonstrated to enhance feed utilization and promote growth in broiler chickens, while also bolstering the immune system and reducing the risk of infection in the host (Nalunga et al., 2021). The microflora present in the host intestine plays a vital role in improving growth and digestion by secreting digestive enzymes and promoting the production of digestive enzymes. They also help inhibit the growth and adhesion of pathogens, preserve the integrity of the gut flora and epithelial barrier, and regulate the immune reaction and antioxidative status to boost the host's immunity to ailment. Additionally, incorporating a combination of multiple probiotic strains (referred to as mixed or multi-strain probiotics) can result in synergistic effects that offer greater advantages to the host organism (Magee et al., 2022).

In recent years, there has been growing interest in the use of probiotics as a potential alternative to antibiotics in the Iranian poultry industry. With its position as the largest in the Middle East and ranking fourth in Asia, the Iranian poultry industry has emerged as a major player in the global market, currently holding the 17th position worldwide. Over the past ten years, this industry has experienced remarkable growth, transforming from small-scale backyard operations to a thriving multibillion-dollar enterprise. In 2022, the global production of poultry meat reached an impressive 145 million tons, with Iran making a substantial contribution to this global figure (Khoshgoftar Manesh et al., 2020). Probiotics, consisting of beneficial microorganisms, have shown promising effects on growth promotion, digestion enhancement, and intestinal health improvement in broiler chickens. However, limited research has focused on the effects of double-strain probiotics, which combine multiple strains of beneficial bacteria.

The cost-effectiveness of these probiotics stems from their widespread availability and practical application in the poultry industry. *Enterococcus faecium* and *Streptococcus thermophilus* strains are commercially produced and marketed as probiotic additives specifically formulated for poultry. Due to their popularity and demand, these probiotics are manufactured in large quantities, leading to economies of scale and affordable pricing. Additionally, the accessibility and availability of these probiotics are facilitated by the well-established distribution networks of agricultural suppliers, making them readily accessible to poultry farmers and integrators. Moreover, the availability and accessibility of *Enterococcus faecium* and *Streptococcus thermophilus* probiotics are further supported by their regulatory approval and inclusion in industry guidelines. These probiotics have undergone thorough evaluation for safety, efficacy, and quality control, meeting the necessary regulatory standards for use in animal feed. Their inclusion in industry guidelines and recommendations further reinforces their acceptance and availability as probiotic options for broiler chicken production.

The viability of probiotics added to block and crumble feed can be compromised by the high pressures and temperatures used during compression, which can create complicated conditions that are incompatible for the viability of probiotics. This can lead to issues with the functionality and dosage of probiotics-supplemented feed (Rocha et al., 2022). At present, probiotics are frequently safeguarded through pre-treatment methods like coating and encapsulation to mitigate losses during the processing stages. Alternatively, probiotics may be manually sprayed onto feed pellets after processing to enhance their survivability (Liu et al., 2023). Although these methods can be efficacious in enhancing the viability of probiotics, they require extra industrial stages and increase costs. The novel method of premixing-spraying was a technique used in this study to add temperature-sensitive supplements like enzymes, beneficial bacteria, and vitamins to animal feed. This technique involves spraying the additives onto the surface of the feed before packaging, which plays a key role in minimizing casualties of beneficial microorganisms during processing and maintaining the effectiveness of the additives. This approach offers several advantages, including improved probiotic distribution, minimizing the loss of microorganisms and adherence to feed particles, leading to enhanced bioavailability and effectiveness. The premix-spray method has gained attention for its potential in improving growth performance, digestion, and intestinal health in broiler chickens. However, this method has not been previously reported for supplementing probiotic cultures. This study examined the effectiveness of the premixing-spraying method for *Streptococcus thermophilus* and *Enterococcus faecium* strains, as well as the impact of nutritional fortification with duo-strain probiotics (DSP) on the growth rate, feed digestion, antioxidant activity, and Gut microbiome of Plymouth Rock. These findings may provide a viable method for incorporating probiotics into the poultry feed industry.

## Materials and methods

### Materials

The duo-strain probiotics (DSP) including *Streptococcus thermophilus* (strains LMG 19221, CMRZ1107) and *Enterococcus faecium* (strains LMD11, MO-ZIW-021) were isolated from Kefir (a fermented milk drink that is similar to yogurt) and conserved in the fridge at –5 °C in the protein supplements lab of UTB Institute (Tehran, Iran). The *S.thermophilus* and *E.faecium* were activated in Tryptic Soy Broth (TSB, Hayan, Iran) and MRS (Thermo, USA) at 36 °C for 12 h. Following coincubation, the mixed cultures were incubated together at 36 °C and 120 rpm in a 20 L molasses-based medium for 48 h to allow for microbial propagation. The results of colony-forming unit (CFU) counting revealed that the probiotic culture contained 2.19 × 10^10^ CFU/mL of *S. thermophilus* and 1.55 × 10^11^ CFU/mL of *E. faecium.*

The dietary treatments were CG (Basal diet), and DSP (CG + 0.5 % supplement). A nutritious diet during early growth (days 1–14), puberty (days 15–21), and final (days 22–30) stages of preparation were planned to meet the provisions of NRC (Table 1). The preparation of DSP was dispersed onto the surface of the basal diet at 0. MPa and a ratio of 1/500 (medium/ diet) using post-spraying apparatus (SprayTech Innovations, Model ProMist 2020, ACEquip Co, Iran). The laboratory procedure for this preparation followed the guidelines outlined in the users' guide manual provided by the equipment manufacturer. Treatment diets were coated with the final concentration of DSP at 10^7^ colony-forming units (CFU)/g. All diets were prepared at Persis Co., (Tehran provance, Iran) and stored at ambient temperature (27.3 ± 2.5 °C) until utilized.

**Table 1:** Nutrient content of broiler feed (as fed).

Item	Starter (1–14)	Grower (15–21)	Finisher (22–31)
Ingredient (g/kg)			
Pepton from casein	10.1	11.6	11.52
Soybean meal	58.62	57.2	56.41
Meat extract	18.9	16.9	18.1
Yeast extract	14.72	17.56	21.9
D(t) – Glucose	20.63	21.43	24.6
K_2_HPO_4_	2.23	2.99	3.1
Tween 80	1.74	1.95	2.1
(NH_4_)_2_HPO_4_	2.39	2.44	2.12
Sodiym chloride	5.56	5.74	4.91
Sodium acetate	5.12	5.88	5.32
Wheat bran	2.08	2.33	2.96
Limestone	12.6	11.8	12.51
Peptic digest of soybean meal	3.44	2.44	3.47
Methionine (99 %)	16	15.07	15.6
Dextrose	2.5	3.4	3.6
Casein peptone	10	10.2	9.81
Lithium chloride	5.02	6.21	5.66
Sodium pyruvate	10.1	11.3	12.7
Glycine	12.6	11.2	13.49
Metabolizable energy (kcal/kg)	3416	3430	3435

Premix provided per kilogram of diet: vitamin A, 13,500 IU; vitamin D3 3600 IU; vitamin E, 25.40 IU; vitamin K3, 3.45 mg; vitamin B1, 4.8 mg; vitamin B2, 5.30 mg; vitamin B6, 6.80 mg; vitamin B12 (cobalamin), 0.05 mg; choline chloride, 320 mg; Fe (from ferrous sulfate), 60 mg; Cu, 8 mg; Mn (from manganese sulfate), 210 mg; pantothenic acid, 16.56 mg; Zn, 55 mg; Iodine (from calcium iodate), 1.1 mg; Se, 0.6 mg; menadione sodium bisulfate, 2.5 mg; niacin, 44.55 mg, thiamin, 2.2 mg; riboflavin, 8 mg; nicotinamide, 40 mg; calcium pantothenate, 10 mg; HCl, 4 mg; Zn, 72.76 mg; biotin, 0.04 mg; folic acid, 1 mg.

### The ability of the DSP to survive in the culture, aerosolization equipment, and feed

The viability of *Streptococcus thermophilus* and *Enterococcus faecium* was assessed using the plate count method. In summary, culture samples were collected before and after exposure to the nozzle of the equipment for premixing-spraying. The samples were then diluted in sterile 0.7 % PBS, plated in duplicate onto MRS and TSB medium, and incubated at 36 °C for 12 h. Colonies on Petri dishes with a bacterial number varying between 10 and 100 were chosen for the estimation of the logarithmic value of viable probiotic cells, using the colony-forming unit (CFU) method. The diet, impregnated with DSP, was stored at ambient temperature (27 ± 2 °C) following the spraying process. For the experiment, 8 g of the DSP-impregnated diet were collected every 2 days, and then suspended in 150 mL of sterile PBS. The suspension was soaked for 20 min and agitated at 180 rotations per minute (r/min) in an incubator for 20 min to make ready a bacterial broth. The bacterial suspension was then subjected to five-fold serial dilution to determine the logarithmic CFU.

### Experimental design

This study was carried out at the poultry farming section situated in Alborz (Qazvin, Iran), in compliance with the approval (No: VBD-5-2023) of the animal ethics review board of Tehran university (Tehran, Iran). Prior to commencing the trial, the rearing houses and equipment underwent thorough disinfection. A total of 360 one-day-old mixed-sex Plymouth Rock chicks, weighing an average of 51.33 ± 0.86 g (mean ± SD), were randomly assigned to one of two treatment groups (DSP and CG). The chicks were obtained from AsiaNovin hatchery (Qazvin, Iran) and placed in 9 replicated cages, each housing 20 chicks. The chicks were raised in a completely randomized, multi-layered protective cage for a period of 31 days.

The keeping temperature of the chickens in the first week was about 32 to 35 °C (relative humidity; 40 % to 70 %) and as the feathers grew, the room temperature decreased every week so that for the second week the room temperature reached 30 °C, in the third week 26 °C, the fourth week 23 °C, and finally from the fifth week onwards the surrounding temperature ranged from 13 to 16 °C. At night, when bird activity decreases, the need for high ambient temperatures is felt, but during the day when there is more activity and food intake, the need for heat was less felt. Therefore, the keeping temperature of adult chickens was kept at around 16 °C at night and between 12 °C and 13 °C during the day. The poultry's body temperature ranged from 40.6 to 41.7 °C. Proper ventilation had been installed for the health and well-being of the chickens during the experimental process. Air flow was constant throughout the aviculture to avoid hot spots or stagnant areas (0.76 km/h), so that the air moved in a circular pattern to ensure that all areas were well ventilated. Each hen had at least 0.3 m^2^ of indoor space and 0.92 m^2^ of outdoor space, and their living quarters were regularly cleaned to prevent the accumulation of debris, bacteria, and parasites.

### Method

Fresh water and feed were ad libitum. Following the initial measurement of the basal diet, the probiotic was supplemented into the feed at a concentration of 0.5 %, exclusive of the control group. The broilers were provided with feed and clean water for 30 days at predetermined intervals until the conclusion of the experiment. This study was tested with dose(s) of 0.3 %, 0.7 %, 1.5 %, and 1.75 % of the supplement.

To assess food intake, the broilers were weighed on days 8, 22, and 31, and the quantity of feed consumed and orts in each coop was documented daily. On day 31, measurements were taken for broiler weight (BW), feed consumption (FC), feed efficiency ratio (FER), and mortality. To measure nutrient digestibility, an indigestible marker, polyethylene glycol (PEG) at a concentration of 0.5 %, was incorporated to the broiler diet on day 22 and administered for a duration of roughly 7 days, until the experiment's conclusion. Representative feed samples were accumulated from each treatment group immediately after mixing the marker, using sterilized plastic bags. On day 31, raw excrement samples were collected randomly from 40 broilers in each treatment group (4 chicks per cage) using a glass tray. The droppings samples were consolidated and delivered to the laboratory, where they were stored at −7 °C for analysis of pure animal feed (PAF), nitrogen (N), and unrefined energy (URE) nutrient digestibility. Before conducting the analysis, the freeze-dried samples were positioned inside an INTBUYING Digital Forced Air Convection Desiccator Heat Industrial Lab, heated at 100 °C for 8 h, milled, and sifted using a 0.5 mm sift. PAF and N approaches were conducted according to the AOAC (2005) method. URE was examined using Isoperibol oxygen bomb calorimetry (BK-1A-BIOBASE Instrument Company, Shandong, China), while N was evaluated using automated Kjeldahl analyzers (multi-EA 5100 C, N, S, X in ONE Analysis Cycle, Analytik Jena US LLC, Upland, CA 91786 USA). PEG absorption was determined using UV-1900i uv-vis spectrophotometry. The digestibility of the whole device was calculated using the formula below:

(1) $$TTD\left(\%\right)=100-\left[\left[\left(\frac{NF}{ND}\right)\times \left(\frac{PEG\;D}{PEG\;F}\right)\right]\times \;100\right]$$

where, TDD, NF, ND, PEG F, and PEG D refer to total tract digestibility, nutritive substance content in the fecal samples, nourishment content in the diet, PEG content in the fecal sample, and PEG levels in the diet, respectively. On the 31st day, fecal samples (40 chickens per treatment) were obtained using a glass tray. The samples were combined, placed in sterilized microtubes at an arranged time (3:00 p.m.), stored in an ice-filled vessel, and promptly transported to the laboratory. A 2-gram portion of fresh fecal sample was collected and mixed with 10 mL of 0.5 % phosphate-buffered saline (PBS) using a vortex mixer. Microbial analysis was performed in accordance with the procedure outlined by Mirsalami and Mirsalami (2024a); Wei et al. (2013). At approximately 6 p.m., fresh fecal samples weighing around 250 g were randomly obtained from 40 birds. The samples were then stored in a 3 L sealed plastic container with a small opening on one side. The container was sealed tightly with adhesive tape and fermented at a temperature of 27 °C for a period of 6 days. On the 7th day, a 150 mL specimen was extracted from the air gap (3 cm) to allow for proper air circulation, after which the container was resealed. The sample container was manually shaken for approximately one minute to determine the presence of any crust formation on the surface. Ultimately, CO_2_, CH_₃_COOH, H_2_S, NH_3_, and CH_4_S were measured using the scopes of 10 to 90 ppm (0.1–20 parts per million (ppm); Gastec detector tubes. NIOSH, USA) and 2.0 to 20.0 ppm (range of 0–20,000 ppm, Dräger detector tubes; Kyushu, Japan).

The Plymouth Rock Broilers (40 chicks/treatment) (4 chicks/cage) were slaughtered through cervical dislocation. The chicken wings, liver, gizzard, milt, drumstick, and pectoral muscle were extracted by the specialists. The tissues were individually measured and expressed as a fraction of the total body mass. The corresponding samples were transported to the testing center, and the breast fillet was extracted for analysis of its grade. The color characteristics of each sample (surface), including redness, lightness, and yellowness, were measured at specific locations using a commercially available portable chroma meter (BYK-Gardner Color-Check). The acidity level and water retention ability (WRA), meat texture, and shrinkage were determined according to the procedures outlined by Rehman et al. (2020).

### Growth assessment

At the start and conclusion of the study, the growth performance of the broiler chickens was assessed by taking individual measurements of their weight and size. Different growth parameters were determined using the following formula:

(2) $$BWG=\left[\frac{\left(FBW-IBW\right)}{IBW}\right]\left(\text{IBW}\text{and}\text{FBW};\text{initial}\text{and}\text{final}\text{body}\text{weight}\right)$$

(3) $$SGR=\left[\frac{\left(\ln FBW-\ln IBW\right)}{Days}\right]\left(\text{SGR};\text{specific}\text{growth}\text{rate}\right)$$

(4) $$SR=\left[\frac{N_{fb}}{N_{ib}}\right](N_{\text{fb}}:\text{Number}\text{of}\text{final}\text{broilers},N_{\text{ib}}:\text{Number}\text{of}\text{initial}\text{broilers})$$

(5) $$FCR=\left[\frac{P_{DSP}}{WG}\right](P_{\text{DSP}}:\text{Probiotic}\text{or}\text{Dry}\text{feed}\text{consumed},\text{WG}:\text{weight}\text{gain})$$

(6) $$CF=\left[\frac{W}{L^{3}}\right]\left(\text{CF};\text{condition}\text{factor}\right)$$

### Assessment of antioxidant capacity

The serum and breast muscle samples were defrosted at a temperature of 27 °C following their retrieval from the cryogenic substance storage facility. The defrosted samples were subsequently sliced into smaller pieces, measured, transferred to sterile glass tubes, and blended with chilled NaCl solution in a frigid water immersion at a suitable weight-to-volume ratio (1:10 for liver and 1:5 for muscle, respectively) until they achieved a uniform consistency with no discernible particulate matter. A mechanically operated homogenizer (Model XYZ-500, ABC Corporation, Arizona, USA) was used for this purpose. The resulting mixture was then subjected to centrifugation at 3580 × g at 5 °C for half an hour. The liquid portion above, supernatant, was collected, divided into labeled 300-μL aseptic cylindrical tubes, and preserved at −45 °C for the analysis of oxidizing agent/reducing agent indicators, such as total antioxidant capacity (TAC), catalase (CAT), superoxide dismutase (SOD), glutathione peroxidase (GPx), protein carbonyls (PCO), and malondialdehyde (MDA).

The antioxidant capacity of broiler serum and midgut samples was assessed with commercial colorimetric test kits at the specified wavelengths, following the methodologies outlined in (Zha et al., 2023) with a little modification. TAC level serves as a delicate gauge of the total cellular antioxidative activity, and as per this study, it was assessed using a widely applicable method where an alternative analytical reagent, such as dinitrophenylhydrazine (DNPH), could be employed. In this method, the existence of intracellular antioxidants leads to the reduction of the DNPH reagent, resulting in the generation of a distinctive color indicative of TAC activity. The conventional hydroxylamine assay (Park & Kim, 2018) was conducted to quantify SOD levels in both serum and tissues. The activity of SOD was determined by measuring the amount necessary to achieve a 60 % reduction in the rate of nitrite production per unit volume of serum or per unit mass of tissue protein content over a 30-minute period at a temperature of 36 °C. One unit of SOD activity was determined based on these criteria. For the quantification of GPx activity, an alternative well-established technique called the Ellman's assay or the DTNB method (Ellman, 1959) was utilized. In this method, 1 unit of GPx activity was determined as the quantity of enzyme necessary to consume 2 micromoles of glutathione in a volume of 150 µL of serum or in a protein amount of 2 mg from tissue samples within a time frame of 10 min at a temperature of 36 °C. High-performance liquid chromatography (HPLC) was executed following the method described by Candan and Tuzmen (2008) to ascertain the MDA level in serum, hepatic, and pectoral muscles as a measure of lipid peroxidation, which was quantified as nmol/mL for serum samples and nmol/mg protein for tissue samples. Prior to statistical comparison, all obtained findings in the liver and primary breast muscle were normalized to their respective overall protein concentrations. The PCO level was assessed using the analytical technique described by Yan and Forster (2011), using the Pierce™ BCA Protein Assay Kit (Thermo Fisher Scientific, Catalog No. 23227)

### Assessment of digestive enzyme activity

The activity of digestive enzymes in the crop, gizzard, duodenum, jejunum, and ileum of broiler chickens was assessed. The intestinal samples, collected in sets of three from each cage, were finely chopped and blended with 0.7 % PEG at a proportion of 1:8 (weight/volume). The resulting mixture was then subjected to centrifugation at 2200 r/min for 20 min at a temperature of 5 °C, and the liquid portion recovered after centrifugation was stored at −17 °C for deeper exploration. The enzymes measured included amylase, protease, and lipase.

Amylase activity was measured using a commercial assay kit (EnzyChrom™ α-Amylase from BioAssay Systems). The assay was based on the hydrolysis of C_24_H_28_ClNO_14_ to liberate C_6_H_4_ClNO_3_, which was measured spectrophotometrically at 405 nm. Protease activity was measured using a commercial assay kit (SensoLyte® Rh110 from AnaSpec). The assay was based on the hydrolysis of N-benzoyl-l-arginine-p-nitroanilide (BAPNA) to liberate p-nitroaniline (PNA), which was measured spectrophotometrically at 405 nm. Lipase activity was measured using a commercial assay kit (QuantiChrom™ from BioAssay Systems). The assay was based on the hydrolysis of p-nitrophenyl laurate to liberate p-nitrophenol (PNP), which was measured spectrophotometrically at 405 nm.

### Intestinal morphology

The portions of the gastrointestinal tract were fixed in paraffin, and each sample segment was mounted on a plastic slide and subjected to hematoxylin-eosin (HE) staining. The villi were examined using a Leica DM4000B microscope (ECHO, San Diego, CA 92126) equipped with the Zenith Image Analysis System. Villus height was quantified as the distance from the highest point of the intestinal protrusion (villus) to the villus-crypt interface, while crypt profundity was measured as the extent of the groove between neighboring villi. All determination procedures and preparation were conducted following the methodology outlined by Mirsalami and Alihosseini (2021) with a little modification.

### Cecal SCFAs analysis

In line with the approach described by Deng et al. (2023), the cecum digesta sample underwent processing to obtain the supernatant. Subsequently, the supernatant was subjected to analysis using a capillary column gas chromatograph (Model ABC-2000; ACME Instruments, London, UK) containing a capillary column (Restek Rxi-5Sil MS; 30 *m* × 0.25 mm × 0.25 μm, Restek Corporation, Bellefonte, Pennsylvania, USA). The concentrations of acetic acid, propionate, butyric acid, valeric acid, butyrate, and isovaleric acid were then determined and expressed as mmol per gram of wet cecal digesta.

### G*enomic DNA isolation, high-throughput sequencing analysis, PCR amplification*

The QIAamp DNA Stool (Pro DNA Isolation Mini Kit from MP Biomedicals) was used to extract total DNA from the intestinal samples following the manufacturer's protocol. The extracted DNA was analyzed for quality and quantity using a NanoDrop spectrophotometer (M200 PRO NanoQuant from BioTek).

PCR replication of the microbial 16S rRNA gene's *V3-V4* zone was accomplished using the primer pair 341F (5′-CCTACGGNGGCCWGCAG-3′) and 806R (5′-GGACTACHVGGGTTATCTAAT-3′). The polymerase chain reaction (PCR) was conducted using a specialized reagent (KOD Xtreme Hot Start DNA Polymerase from Merck). The AxyPrep Gel Extraction Kit was utilized to purify the PCR products (Wizard SV Gel and PCR Clean-Up System from Omega Bio-Tek).

The PCR products that underwent purification were sequenced through the use of PacBio sequencing Systems (PacBio Inc., Menlo Park, CA 94025 USA). The raw sequencing data were processed using the QIIME2 pipeline (v2021.4). The DADA2 plugin was utilized to execute quality filtering, denoising, and chimera removal processes. The taxonomy assignment was performed using the SILVA database (version 138) and the feature-classifier plugin. The *α* and *β* diversity metrics were computed using the q2-diversity plugin.

### Statistical analysis

To ensure clarity and reproducibility, we conducted a thorough scrutiny of the data for errors. The nature of our data was determined to be non-parametric. As such, we employed appropriate statistical techniques to analyze the data robustly and independently, enabling our work to stand alone. The General Linear Model (GLM) technique from the SAS platform (SAS Institute, Cary, NC) was utilized for the analysis. In cases where significant differences were observed among treatment means, post-hoc tests (specifically, Tukey's HSD) were conducted to determine specific pairwise differences between treatments. The birdhouses served as the experimental units, and the standard error of the means was utilized to express the variability in the data. By adhering to these rigorous statistical procedures, we ensured that our findings were accurate and reliable. A P-value threshold of less than 0.05 was used to determine statistical significance.

## Results

### The potential for the DSP to survive and thrive in both culture and diet

**Fig. 1:**
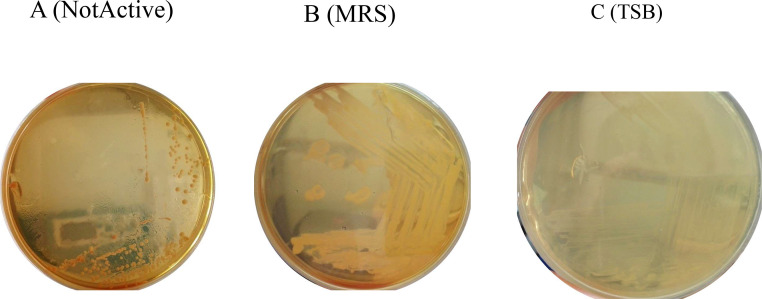
The impact of temperature change on DSP strain prior to spraying was investigated. The following were evaluated: (A) Combination of S. thermophilus and E. faecium strains at 30 °C, where microorganism growth is inactive at or below 30 °C. Meanwhile, (B) and (C) bacterial growth on MRS and TSB medium, respectively, were assessed at 37 °C and pH levels ranging from 6.5 to 7.

**Fig. 2:**
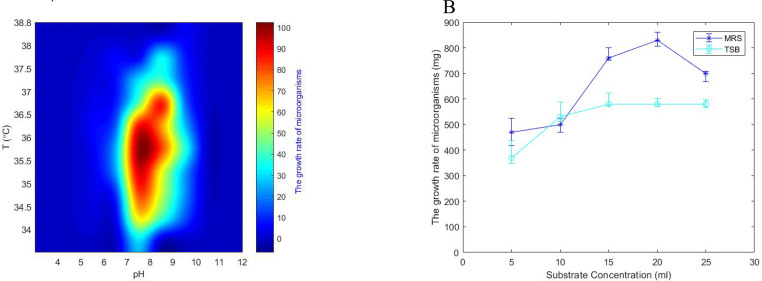
The impact of concentration, pH level, and temperature operational variables on the growth rate of the DSP complement within a 48-hour timeframe. (A). The effect of variations in temperature and the specific characteristics of the culture medium (whether it is basic, acidic, or neutral) on the rate of growth of the DSP strain composition. (B) The effect of optimal concentration of DSP and the effectiveness of strain in protecting the survival of probiotic during the culture period.

**Fig. 3:**
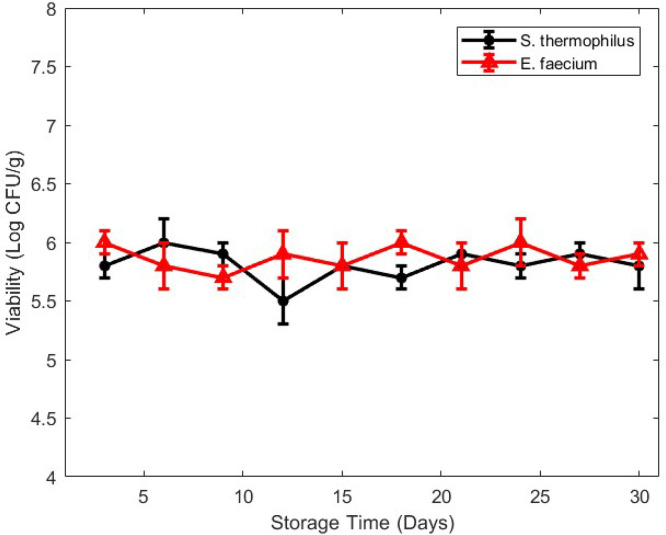
The effectiveness of the dietary DSP when stored at room temperature (27 ± 2 °C). The reported values represent the average ± standard deviation of log colony-forming units per gram for three independent trials.

The results of our study showed that the growth of the microorganism was temperature-dependent, with a notable increase in multiplication rate as the temperature rose. The strain exhibited different growth rates in different culture media. Additionally, the growth rate of the strain was influenced by pH and substrate concentration. When applying the DSP to the diet using the post-spraying method, the viability of the probiotics remained high between the distribution of rations and culture as well as between the distribution of rations and nutrition. During storage at room temperature, the viability of the probiotics stabilized with minor fluctuations over the course of one month. The viability of *S. thermophilus* and *E. faecium* was measured during storage, showing initial increases followed by slight decreases and eventual stabilization. At the end of the one-month storage period, the viability of both strains had decreased slightly. These findings demonstrate the temperature sensitivity of the microorganism's growth, the influence of culture media, pH, and substrate concentration on growth rate, as well as the viability of probiotics during distribution and storage. Additional details and specific numerical values can be found in Table 2 and Figs. 1, 2, and 3.

**Table 2:** Impact of premixing-spraying technique on probiotic survival.

Strain	CMBS (log CFU/mL)	CMAS (log CFU/mL)	DSPSD (log CFU/mL)	pH	culture medium
*S.thermophilus*	10.31±0.5^a^	11.06±0.4^b^	10.05±0.7^ab^	7.1–7.8	MRS
*E. faecium*	10.66±0.7^a^	10.98±0.7^b^	10.57±0.2^ab^	7.1–7.6	MRS
*S. thermophilus*	9.86±0.4^a^	9.74±0.5^b^	9.43±0.4^ab^	7.2–7.4	TSB
*E.faecium*	9.31±0.1^a^	9.02±0.5^b^	9.81±0.8^ab^	7.2–7.5	TSB

Results are presented as the mean ± standard deviation of log CFU/g for 3 replicates, and statistically significant differences between values in the same row are denoted by different superscript letters (*p* < 0.05). CMBS, culture medium before sprayed; CMAS, culture medium after sprayed; DSPSD, duo-strain probiotics supplemented diet.

### Growth rate in different medium

The combination of *S. thermophilus* and *E. faecium* was successfully used as a probiotic and had a positive impact on the host by modulating the digestive system. The treatment diarrhea performance and weight gain of young chicken fed bacterial strain supplement is reported in Table 3. Including probiotics at a level of 0.5 % in the diet appears to have a salutary effect on the BWG of broilers. Specifically, there was a tendency towards increased BWG on the 7th day (*p* *=* *0.068*), and a substantial rise on the 21st day (*p* *=* *0.023*) and entire experimental period (*p* *=* *0.0441*) when compared to broilers on the control diet. The broilers fed 0.5 % DSP supplement in this study did not experience any improvement in feed conversion ratio (FCR) or feed intake over the entire experimental period. The results of our study indicate that adding 0.5 % DSP supplementation to the dietary regimen of broilers did not yield any notable impact on PAF, nitrogen, or URE levels, as shown in Table 4.

**Table 3:** Influence of DSP dietary supplement on broiler growth and performance.

items	CON	DSP^1*^	SEM^2⁎^	*p-*value
First week				
BWG	144	175	8.05	0.064
FI	161	201	5.14	0.425
FCR	1.12	1.15	0.03	0.947
Second week				
BWG	580	655	5.86	0.022
FI	767	881	17.93	0.334
FCR	1.32	1.34	20.2	0.246
Third week				
BWG	897	963	29.7	0.442
FI	1805	1927	44.71	0.489
FCR	2.17	2.01	0.07	0.705
Fourth week				
BWG	1576	1847	29.33	0.071
FI	2881	3105	51.92	0.249
FCR	1.82	1.68	0.08	0.471
SR	4.33	6.82	1.89	0.620

Body weight gain (BWG), survival rate (SR), and feed conversion ratio (FCR), feed intake (FI). DSP^1^*: S. thermophilus and E. faecium composition; SEM^2^*: Standard error of means.

**Table 4:** Impact of DSP supplement on broiler nutrient absorption.

items	CON	DSP^1*^	SEM^2⁎^	*p-*value
N	56.12	57.44	0.58	0.138
PAF	63.74	64.89	0.42	0.027
URE	66.32	65.75	0.38	0.342

DSP1*: S. thermophilus and E. faecium composition; SEM2*: Standard error of means; pure animal feed: PAF, nitrogen: N, and unrefined energy: URE.

The noxious compounds generated in manure can cause health problems for both farm laborers and livestock. The results of this study demonstrate that including a 0.5 % dose of probiotic supplement in the diet of broilers led to a significant reduction in H_2_S and CO_2_ emissions, while nitrogen oxide (N_2_O) remained almost unaffected, the parameters of NH_3_ and methane (CH_4_) recorded a decrease (as illustrated in Table 5). The current investigation shows that incorporating a 0.5 % composition of *S. thermophilus* and *E. faecium* in broiler feed resulted in a substantial increment in the Gut health index (*p* *=* *0.054*), a decrease in *E. coli* counts (*p* *=* *0.066*) and had no discernible effect on *Salmonella* counts (as demonstrated in Table 6).

**Table 5:** Influence of DSP dietary supplement on broiler gut gas emissions.

Items (ppm)	CON	DSP^1*^	SEM^2⁎^	*p-*value
Methane (CH_4_)	1829.7	1656.4	2.87	0.366
CO_2_	1936	1420	242.2	0.523
Nitrous oxide (N_2_O)	213	210	11.4	0.471
H_2_S	4.5	1.01	1.88	0.227
Ammonia (NH_3_)	16.5	13.3	2.94	0.546

DSP^1*^: S. thermophilus and E. faecium composition; SEM^2⁎^: Standard error of means.

**Table 6:** Effect of DSP supplementation on excrements microbiota of broilers.

Items (log_10_CFU/g)	CON	DSP^1*^	SEM^2⁎^	*p-*value
*Salmonella*	6.23^ab^	5.95	0.118	0.425
*E. coli*	6.55^ab^	6.04^ab^	0.04	0.066
Gut health index	10.72	13.88	0.07	0.521

DSP^1*^: S. thermophilus and E. faecium composition; SEM^2⁎^: Standard error of means. The means within the same row that are labeled with different letters (a and b) are significantly different (*p* < 0.05).

The quality of chicken meat treated with probiotics was not significantly different in its taste compared to the control group (CG). However, consumer preferences may influence the taste of chicken meat treated with probiotics. Some consumers may prefer the taste of chicken meat treated with probiotics, while others may prefer the taste of chicken meat from the control group. The results of relative body weight (%) are as follows; the pectoral muscle exhibited a 2.8 % increase in relative weight compared to the control group. The liver and spleen experienced an increase in relative weight, rising from 4.51 % and 0.78 % to 5.23 % and 0.89 %, respectively. Similarly, the relative weight (%) of other organs, such as abdominal fat and gizzard, increased from 2.07 % and 4.19 % to 2.18 % and 4.88 %. Notably, the Bursa Fabricius and Drumsticks displayed a nearly identical increase of 2 %. In summary, the results observed in this study show that the DSP combination resulted in an enhancement of the development of pectoral and femoral muscles compared to the basic diet. As anticipated, the moisture content of the meat exhibited a consistent pattern during storage for both the treatment and control groups. On the initial day, the moisture content was 4.98 and 4.02 for the control and treatment groups, respectively, which then increased to 7.31 and 6.98 after two days. Eventually, the moisture content of the meat reached 18.47 and 17.51 for the treatment and control groups, respectively, compared to the initial levels of 13.69 and 13.07. Briefly, meat moisture was maintained by reducing drip loss, which contributed to excessive meat moisture loss during storage and processing. However, the flavor and aroma of meat in broilers fed with DSP did not show significant changes, as shown in Table 7. Thus far, there remains a lack of comprehensive understanding regarding the impact of incorporating 0.5 % DSP as a supplement in broiler diets on meat quality. It is imperative to conduct further investigations encompassing various doses of DSP to thoroughly assess their effects on the production characteristics of broilers.

**Table 7:** Effect of DSP supplementation on meat quality and limb weight of broilers.

Items	Treatments			
	CON	DSP^1^	SEM^2^	*p*-value
Relative organ weight (%)				
Pectoral muscle	23.4	25.11	0.97	0.865
Liver	4.51	5.23	0.45	0.712
Spleen	0.78	0.89	0.14	0.912
Abdominal fat	2.07	2.18	0.33	0.248
Gizzard	4.19	4.88	0.78	0.331
Bursa of Fabricius	0.86	1.53	0.66	0.419
Drumstick	18.9	20.4	0.47	0.554
Pectoral muscle color				
Redness	13.72	13.95	1.91	0.547
Yellowness	14.88	13.52	2.13	0.682
Lightness	64.91	66.01	1.89	0.791
				
Cooking loss,%	31.04	28.56	2.45	0.186
pH value	5.87	5.43	0.07	0.447
WHC,%	55.47	56.02	5.14	0.871
Drip loss,%				
Day 1	4.98	4.02	0.47	0.317
Day 3	7.31	6.98	0.66	0.442
Day 5	13.69	13.07	0.28	0.501
Day 7	18.47	17.51	0.13	0.233

DSP^1^: S. thermophilus and E. faecium composition; SEM^2^: Standard error of means, WHC: water holding capacity.

### Antioxidant content

**Fig. 4:**
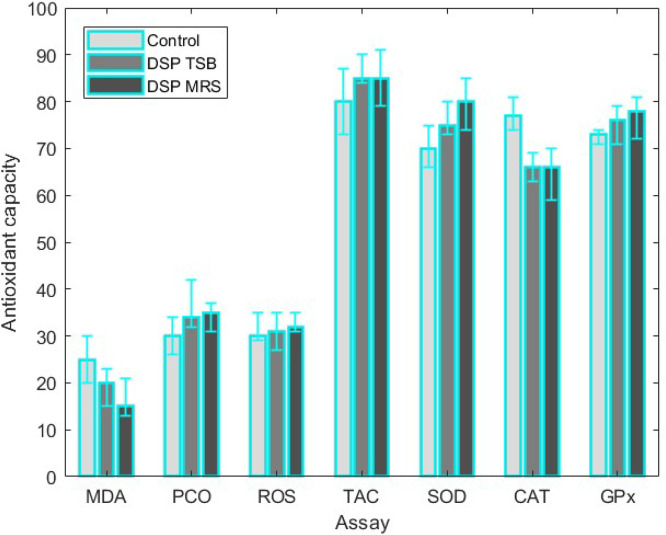
The impact of dry-spraying dietary DSP on the antioxidant capacity of broilers was assessed. The mean ± standard deviation values for the following antioxidant enzymes: MAD, CAT, ROS, PCO, TAC, SOD, CAT, and GPx, were determined for nine independent trials. All values were measured in units per milligram of protein (U/mg protein).

In the DSP groups, the levels of GPx, SOD, and CAT activity were notably elevated, while the levels of PCO and MDA were substantially diminished when compared to the CG (*P <* 0.05). However, there was no meaningful difference in the amount of ROS between the DSP and control groups in either the serum or midgut samples. TAC showed that DSP supplementation improved antioxidant levels in serum or midgut samples compared to the CG. The FRAP assay showed no notable difference in antioxidant capacity between the probiotic and CG in serum samples (*P > 0.05*). Similarly, the ORAC assay a slight difference in antioxidant capacity between the probiotic and CG in midgut samples (*P > 0.05*). The findings of antioxidant capacity were illustrated in Fig 4.

### The enzymatic activity involved in digestion

**Fig. 5:**
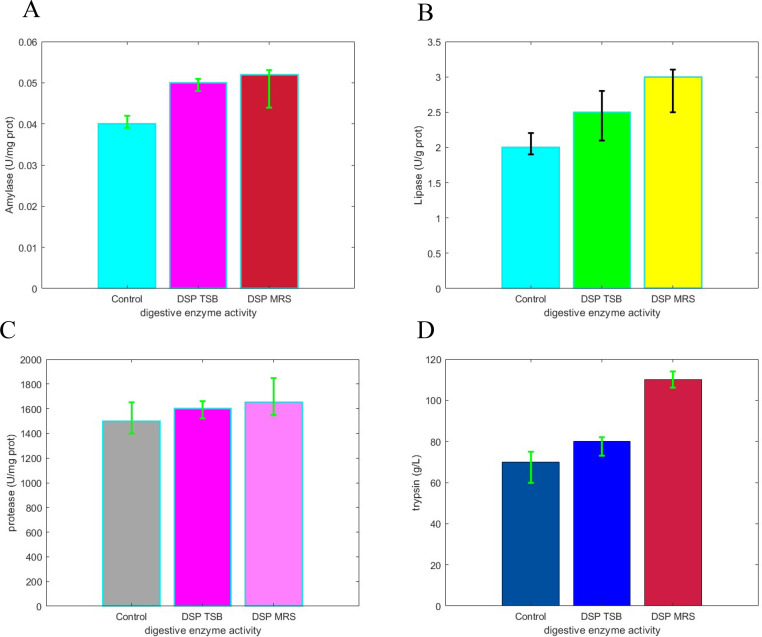
Impact of dietary *E. faecium* and *S. thermophilus* on the activity of digestive enzymes in broilers was evaluated. The mean ± standard deviation values for (A) amylase, (B) lipase, (C) protease, and (D) trypsin were determined for nine independent trials, and significant differences (p < 0.05) between groups were indicated by different letters.

The digestive enzyme activity was measured in the pancreas, duodenum, and jejunum of the chickens, and compared to CG. The results showed that the activity of pancreatic enzymes, including amylase (Fig. 5A), lipase (Fig. 5B), protease (Fig. 5C), and trypsin (Fig. 5D) was notably higher in the probiotic group compared to the CG (*P < 0.05*). Similarly, the activity of duodenal enzymes, including lactase, maltase, and sucrase, was significantly higher in the probiotic group compared to the CG (*P < 0.05*). However, there was no substantial difference in the activity of jejunal enzymes between the probiotic and CG.

### Effects of DSP on the intestinal morphology of Salmonella-infected broilers

**Fig. 6:**
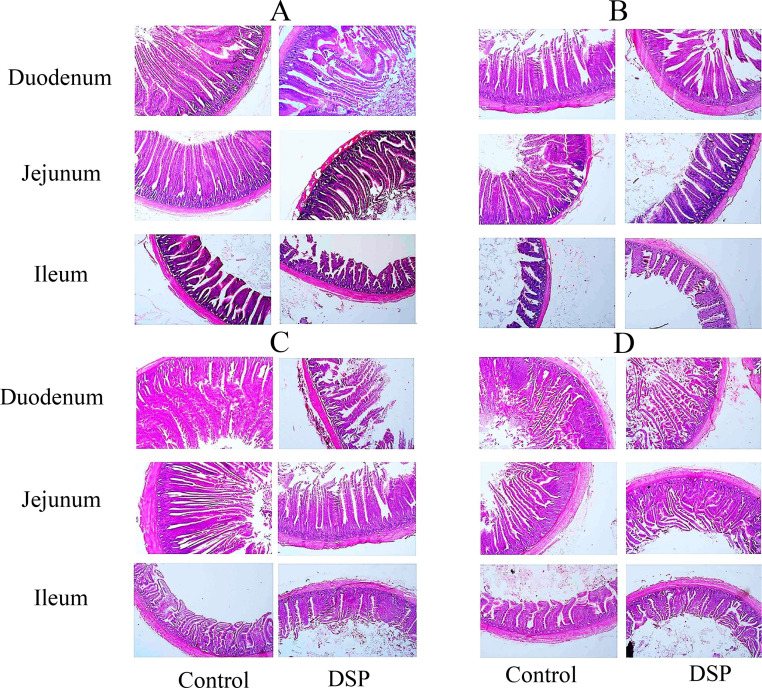
Effects of Enterococcus faecium, and Streptococcus thermophilus on the intestinal morphology of broilers fed diets supplementation, 60Co γ-ray. Control, birds fed with basal diet; DSP, 110 mg/kg DSP. (A) day, 7, (B) day 14, (B) day 21, (C) day 30.

On day 7, histological examination of the Duodenum using HE staining revealed that the DSP treatment resulted in a substantial increase in microvillus elevation (MVE) relative to the CON group (*P* < 0.05). Although the crypt profundity (CP) did not show significant changes across groups, the CON therapy markedly reduced the microvillus elevation / crypt profundity (MVE/CP) ratio (*P* < 0.05), which was effectively restored by the DSP treatment (*P* < 0.05). In the jejunum, the DSP treatment caused a notable increase in MVE (*P* < 0.05) and MVE/CP ratio (*P* < 0.05) compared to the CON group. No substantial effects of the distinct treatments were observed on the assessed morphological parameter in the ileum on the 7th day (Fig. 6A). On day 14, the DSP treatment remarkably enhanced the MVE and MVE/CP amount (*P* < 0.05) of the duodenum compared to the CON group. Additionally, the jejunal and ileal MVE were significantly increased in the DSP group compared to the CON group (*P* < 0.05). Furthermore, in broilers with *Escherichia coli* (E. coli), the jejunal MVE/CP content was reduced compared to the CON group (*P* < 0.05), but the DSP treatment significantly heightened the PVE/CP level compared to the base diet treatment (*P* < 0.05) (Fig. 6B). On days 21 and 30, birds in the DSP group had higher showed higher microvillus elevation / crypt profundity (MVE/CP) level (*P* < 0.05), and enteroendocrine cells number of duodenum and ileum than those in the CON groups (*P* < 0.05). Moreover, birds in the DSP group had higher enteroendocrine cell numbers and microvillus elevation of jejunum than those in the CON groups (*P* < 0.05) (Fig. 6C,6D).

### Cecal microflora community compositions

**Fig. 7:**
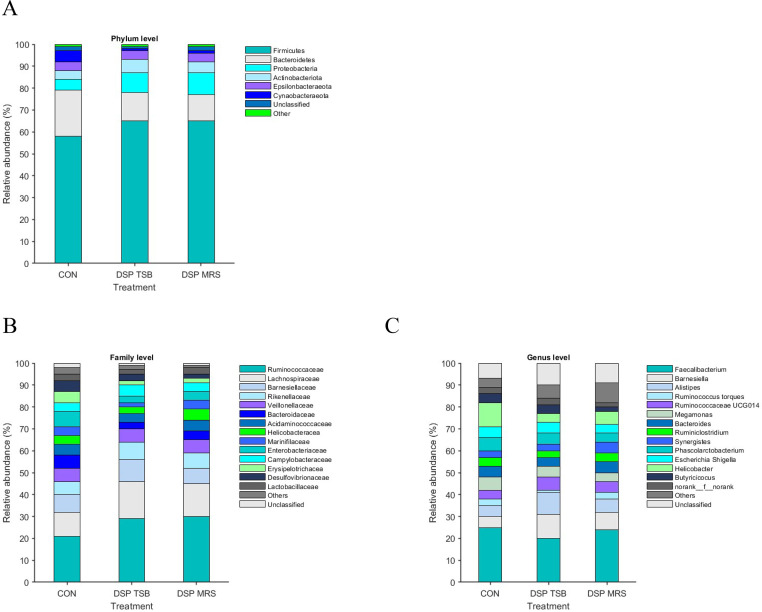
The impact of dietary supplementation with DSP on the cecal microbiota composition of broiler chickens was examined on day 31, focusing on the phylum, family, and genus levels. Microbiota composition at these levels was denoted as (A, B, C) respectively. Statistical analysis revealed a significant difference at *P* ≤ 0.05.

In all samples, the dominant phyla were *Firmicutes* and *Bacteroidetes*, with *Proteobacteria, Actinobacteriota, Epsilonbacteraeota*, and *Cynaobacteraeota* following suit (refer to Fig. 7A). Moreover, the inclusion of DSP led to a considerable reduction in the percentages of *Cynaobacteraeota* and *Bacteroidetes* phyla compared to the CON groups (*P* < 0.05; see Fig. 7A). Concerning the family level, the primary intestinal flora across all samples consisted of *Ruminococcaceae, Lachnospiraceae, Barnesiellaceae, Rikenellaceae, Bacteroidaceae, Veillonellaceae, Enterobacteriaceae*, and *Helicobacteriaceae* (refer to Fig. 7B). However, the abundance of acid-producing bacteria, specifically *Ruminococcaceae* in the cecum of DSP broilers, was considerably higher compared to other groups (*P* < 0.05; see Fig. 7B). When examining the genus level, the two most dominant genera were *Faecalibacterium* and *Barnesiella*, followed by *Alistipes, Helicobacter, Ruminococcus* UCG014, Phascolarctobacterium, Others, and unclassified (refer to Fig. 7C). Additionally, the inclusion of DSP-TSB resulted in a notable increase in the beneficial bacteria *Alistipes* compared to the CON and DSP-MRS groups (*P* < 0.05; see Fig. 7C).

### Intestine microbial diversity

**Fig. 8:**
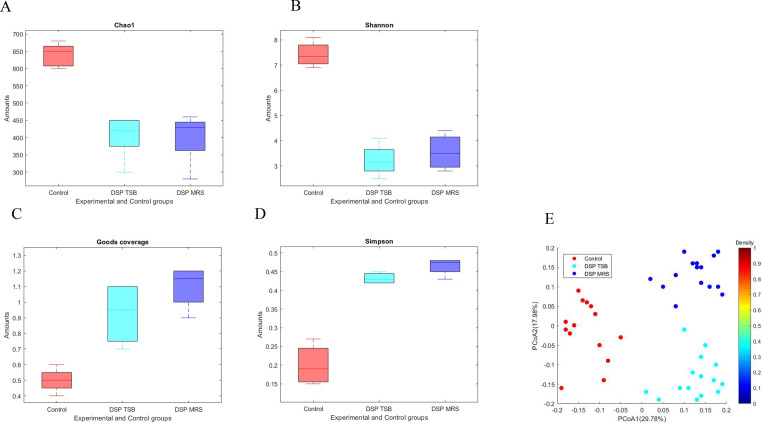
The impact of dietary DSP on the diversity of intestinal microflora in broilers was examined through assessment of both *α* and *β* diversity. Alpha diversity was measured using four indices: (A) Chao1, (B) Shannon, (C) Goods coverage, and (D) Simpson, while (E) *β* diversity was analyzed utilizing PCoA.

The results revealed no substantial difference in the bacterial diversity between the MRS and TSB culture media. However, a considerable difference was observed in the bacterial diversity of the broiler chickens between the experimental and CG (*P > 0.05*). The Chao1 and Shannon indices were slightly reduced in the experimental group compared to the CG, indicating a slight decrease in species richness and evenness. In contrast, the Simpson and Good's coverage index increased noticeably in the experimental group compared to the CG, suggesting an increase in community dominance and phylogenetic diversity. The diversity of intestinal samples at the alpha (*α*) level was indicated by several indices, including Chao1 (shown in Fig. 8A), Shannon (shown in Fig. 8B), Good's coverage (shown in Fig. 8C), and Simpson (shown in Fig. 8D). Moreover, the beta (*β*) diversity was visualized using a PCA plot (depicted in Fig. 8E), which revealed significant differences between the CG and the DSP-supplemented diet group (P < 0.05), as evidenced by the noticeable separation between the two groups.

The results of this study suggest that the DNA isolation, PCR amplification, and high-throughput genomic sequencing methods used were effective in analyzing the intestinal microbial diversity of broiler chickens. The lack of significant difference in bacterial diversity between the two groups indicates that any potential confounding factors, such as differences in the DNA extraction or sequencing methods, did not significantly affect the results. The slight decrease in the Chao1 and Shannon indices in the experimental group may be due to the differences in diet and housing conditions between the two groups. The increase in the Simpson index and Faith's Phylogenetic Diversity index in the experimental group suggests an increase in the dominance of certain bacterium strains and an increase in the phylogenetic diversity of the microbial community. These changes in community structure may be due to the specific dietary and housing conditions used in the experimental group. Further research is needed to determine the specific factors that influence the intestinal microbial community in broiler chickens and to develop strategies for promoting a diverse and healthy microbiota in these animals.

## Discussion

Probiotics are a preferred replacement for antibiotic use in the poultry industry to reduce the extension of microbial resistance. As regards probiotics are desirable microorganisms with anti-bacterial and growth-stimulating virtues, This Study seeks to recognize and assess the effects of *S. thermophilus* and *E. faecium* species on broiler chickens.

### Exploring the varied effects of probiotic supplementation on broiler performance

Glombowsky et al. found that supplementing broiler diets with *Bifidobacterium* improved body weight gain (Glombowsky et al., 2020). Conversely, Sirovanik et al. reported no effect of probiotic supplementation on broiler body weight gain, which is in contrast to the findings of our study (Sirovnik et al., 2021). While de Franca et al. reported improved feed conversion ratio (FCR) with DSP supplementation, our study found no improvement in FCR or feed intake in broilers fed a 0.5 % DSP supplement throughout the experimental period (de França et al., 2023). To corroborate our findings, Broom & Kogut similarly found that DSP (*E. faecium*, and *Bacillus*) supplementation did not affect feed intake in Indian cockerel (Broom & Kogut, 2018). Therefore, the lack of improvement in feed consumption and feed conversion ratio (FCR) observed in our study may have been due to insufficient levels of energy or protein in the experimental diet, as well as environmental factors. In general, the underlying reasons for the observed lack of nutrient digestibility in Plymouth Rock fed 0.5 % DSP remain unclear. Therefore, further studies are warranted to elucidate the impact of DSP on chicks' nutrient digestibility by modifying the level of supplementation and experimental diet composition. Other factors that can affect feed consumption and FCR include the type of feed, the feeding frequency, and the health of the animals (Musigwa et al., 2020). The observed improvements in BWG in broilers fed 0.5 % DSP-supplemented diets containing *S. thermophilus* and *E. faecium* in our study may have been due to the increased population of beneficial gut flora and elimination of pathogenic microorganisms. A nutrient-rich diet containing appropriate levels of energy and free amino acids (FAA) is crucial for optimizing feed utilization (Liang et al., 2024; Mirsalami & Alihosseini, 2023).

### Probiotic supplementation in poultry: effects on feed consumption, growth performance, and nutrient digestibility

Addition probiotic-supplemented, either as solitary or amalgamation dose of DSP resulted in improved feed consumption (FC), BWG, and Feed conversion ratio gain (FCRG). It is noteworthy, the DSP-supplemented beneficial impacts were more obvious when compared to *penicillin* or its derivatives supplement. The addition of DSP-supplemented probiotics to chicken feed has been found to significantly improve growth performance and reduce FCR even under stressful conditions such as diarrhea and anorexia. In a study by Salehimanesh et al., it was observed that a combination of *L. salivarius* and *E. faecium* composition in probiotic supplementation containing soybean meal was as effective as antibiotic treatment in improving growth parameters in broiler chickens exposed to diarrhea, especially during the initial stage (Salehimanesh et al., 2016).

Likewise, Gorenz et al. observed that feeding Sussex (breed of egg-laying hens) with a probiotic supplement containing *Bacillus coagulans* and *S. thermophilus* did not result in meaningful distinctions in the digestibility of PAF and nitrogen (Gorenz et al., 2022). Similarly, Lee et al. found that including 0.75 % *Bacillus subtilis* (1 × 210 CFU/kg) in the diet of growing Leghorn (a type of laying hen) did not influence the apparent whole tract digestibility of PAF and N_2_ (Lee et al., 2023). In contrast, Carron et al. reported that supplementing the diet of growing Wyandotte with probiotics containing *Pediococcus acidilactici* and *E. faecium* improved nutrient digestibility (Carron et al., 2017), which was also noted by Mikulski et al. (2012). It is possible that the differences in these results are due to variations in probiotic strains or animal species. The impact of *S. thermophilus* and *E. faecium* supplementation at 0.5 % inclusion rate on nutrient digestibility in broilers is unclear, and further studies are required to explore the effect of different supplementation levels and experimental diet compositions.

### Effect of probiotic supplementation on ammonia and odor emissions in poultry

Our findings are consistent with the results of Lu et al., who observed a reduction in H_2_S, NH_3_, and total mercaptans emissions in Plymouth Rock broilers fed a diet fortified with *Bifidobacterium* and *E. faecium* complex (Lu et al., 2023). Likewise, An et al. discovered that supplementing the diet of growing roosters with 0.4 % of a probiotic containing *L. acidophilus* significantly reduced NH_3_ emissions (An et al., 2023). Furthermore, according to Mbaye et al. the addition of probiotics to livestock feed led to a significant reduction in levels of NH_3_, fecal pH, and fugacious organic substance, as well as the elimination of toxic odors (Mbaye et al., 2023).

### Impact of probiotic supplementation on pathogenic microorganisms and intestinal health

In this study, another subject that was examined closely was a pathogenic microorganism that had a significant impact on the health of the animal. *Escherichia coli* (E. coli) is a significant pathogen causing loose stools in animals. Previous research has shown that a diet containing *Bacillus subtilis* can impede the growth of pathogens by competing with them for limited nutrients, adjusting the composition of gut microflora, and establishing a biological barrier (Rousseau & Gomes, 2022). When *E. faecium* and L. *plantarum* supplements are introduced into the intestinal tissue, they could convert sugar molecules into glucose and fructose. Additionally, they can lower the pH of the intestine and enhance the activity of aldehyde, alcohol groups, fats, and trypsin. Consequently, these effects result in the length of the intestinal villi, which is beneficial for the absorption and digestion of poultry food, as noted in the study by Kabir (2009). To corroborate our findings, Qiu et al. reported that administering a probiotic mixture containing *L. acidophilus, Pediococcus acidilactici,* and *Lactobacillus reuteri* considerably improved stool *Bacillus subtilis* colonies and reduced *E. coli* levels in Cobb broilers (Qiu et al., 2021). One likely explanation for the increased body weight gain and reduced levels of H_2_S in feces emissions could be attributed to the existence of beneficial bacteria (*S. thermophilus* and *E. faecium* composition) in the gut of broilers.

### Effects of DSP supplementation on meat quality in broilers

The meat quality characteristics were not influenced by the experimental diet in our study. However, Kriseldi et al. demonstrated that incorporating 0.1 % DSP-groups probiotics, including S. thermophilus (1.0 × 220 cfu/g), in the food intake of Plymouth Rock broilers decreased meat softness and enhanced meat pigmentation (Kriseldi et al., 2017). Nevertheless, in our study, the softness of meat and meat color in broilers fed 0.5 % DSP composition remained unchanged. The effects of supplementing broiler diets with 0.5 % DSP on meat quality have not been comprehensively investigated to date. Further studies with varied doses of DSP-supplemented groups are required to assess their impact on the production traits of broilers.

### Effect of MRS culture medium on immunoglobulin levels and disease resistance

MRS culture medium increases immunoglobulin A, G and M levels in broiler chickens and helps to increase growth performance and sickness resistance. MRS and TSB culture medium impregnated with soybean meal and D(t) Glucose helps to increase food digestibility, maintain optimal intestinal microbiota and promote intestinal integrity against environmental diseases. Therefore, by maintaining all operating conditions and keeping away pathogens that can cause diarrhea or malnutrition in poultry during and after growth, the results of this study can be used to solve the problem of weight loss and infectious diseases among chickens which is quite promising and practical.

## Conclusions

Overall, the DSP dietary supplement, which combines two strains of *S. thermophilus* and *E. faecium* with the specified doses, resulted in the alleviation of diarrhea and enhanced growth performance in chickens. In fact, the spray premix technique incorporating DSP-MRS and DSP-TSB demonstrated a reduction in the mortality rate and exerted a positive influence on BWG, SGR, and FCR. Moreover, it significantly modulated the population of *E. coli* within the broilers' gut microbiota. Furthermore, the combined treatment yielded noteworthy improvements in the duodenum and jejunum tissue morphology over a span of 4 weeks. Specifically, the DSP-MRS and DSP-TSB treatments substantially increased the height of the villi compared to the CON treatment when evaluating the morphological parameters of the duodenum and jejunum, while the ileum exhibited no effect. Our findings indicate that the inclusion of 0.5 % DSP in poultry feed could elevate the relative abundance of *Ruminococcaceae* and *Faecalibacteriumin*, enhance antioxidant capacity, and augment digestive enzyme activity. Taken together, this approach and formulation hold great promise for probiotic food supplementation in Iran's poultry industry. The industry faces challenges in sourcing chicken feed raw materials like soy, corn, and wheat due to the arid climate, limited agricultural land, and the high cost of imported food supplements such as antibiotics and probiotics. However, this approach offers a potential solution, providing a cost-effective alternative to address these limitations.

## CRediT authorship contribution statement

**Seyed Mehrdad Mirsalami:** Writing – original draft, Software, Methodology, Investigation, Formal analysis, Data curation, Conceptualization. **Mahsa Mirsalami:** Writing – review & editing, Visualization, Validation, Supervision, Resources, Project administration, Funding acquisition.

## Declaration of competing interest

The authors declare that they have no known competing financial interests or personal relationships that could have appeared to influence the work reported in this paper.
